# Psychosocial Adjustment Factors Associated with Child–Parent Violence: The Role of Family Communication and a Transgressive Attitude in Adolescence

**DOI:** 10.3390/healthcare12070705

**Published:** 2024-03-22

**Authors:** Ana Romero-Abrio, Gonzalo Musitu-Ochoa, Juan Carlos Sánchez-Sosa, Juan Evaristo Callejas-Jerónimo

**Affiliations:** 1 Department of Education and Social Psychology, Pablo de Olavide University, 41013 Sevilla, Spain; gmusoch@upo.es (G.M.-O.); jecaljer@upo.es (J.E.C.-J.); 2 Faculty of Psychology, Autonomous University of Nuevo León, Monterrey 64460, Mexico; juan.sanchezss@uanl.edu.mx

**Keywords:** child-to-parent violence, adolescence, family communication, psychological distress, positive attitude towards transgression of rules

## Abstract

According to official sources, the amounts of children-to-parent violence (CPV) in most advanced countries have been on an increasing trend for more than a decade, which generates great social concern. This phenomenon has also aroused enormous interest among researchers, who have identified risk and protective factors related to adolescent CPV in numerous studies. The aim of the present study was to analyse the relationship between offensive family communication and CPV in adolescence, and the moderating role that two psychosocial adjustment factors may be playing: a positive attitude towards the transgression of rules and psychological distress. A total of 7787 adolescents between 11 and 16 years of age (*M* = 13.37, *SD* = 1.34) from secondary schools in the state of Nuevo León (Mexico) participated in the study (51.5% boys, 48.5% girls). Structural equation modelling was performed using structural equation modelling software (EQS). The results showed that offensive family communication has a direct and significant relationship with CPV. It was also observed that there is an indirect relationship between both variables, through the relationships of psychological distress and a positive attitude towards the transgression of rules. The multigroup analysis performed showed gender differences in some of these relationships. Finally, the results and their implications in the field of family intervention are discussed.

## 1. Introduction

Child-to-parent violence (CPV) is defined as any physical, psychological, or economic violence repeatedly used by adolescents against their parents (or those acting as such) [1,2,3]. In recent years, the number of recorded cases of CPV in developed countries has risen exponentially, generating major social concern. The prevalence of CPV in Spain shows differentiated data depending on the type of violence. The rate ranges from 4.6% to 22% in the case of physical violence, between 45% and 95% in verbal violence [4,5,6]. In Canada and the United States, the CPV rate stands at 12% and 34% for physical violence, respectively, and 60% and 64% for psychological violence [7,8]. These figures reflect a growing socio-educational problem and in recent years, it has led a wide range of professionals—across legal, educational, health, psychotherapeutic, etc., disciplines—to study the causes of CPV, the main factors involved, and the possible means of prevention and intervention in families.

In the field of psychosocial research, several authors lately have focused on the factors involved in the genesis and development of CPV [9,10,11]. In the present study, we chose to approach CPV based on the ecological model of Bronfenbrenner (1994) [12]. This model suggests that human development is the result of the interaction of the person (their genetic characteristics) with the different contexts of socialization. Both the nearest environments, such as the family or school (microsystems), and the most distant ones, such as the social system or culture (macrosystem) influence the individual. Moreover, the environments are interrelated, forming a very complex network of connections that interact with each other throughout the life cycle of the subject [13]. The ecological approach allows us to analyse the risk and protective factors related to CPV in the different contexts of adolescent socialisation. In this study, the focus is on the family microsystem, as it is one of the most influential in the development of the subject at this stage. 

In the family context, several studies have observed that parental socialisation styles are related to CPV. Among these styles, the authoritarian style is the style that is the most strongly associated with greater psychosocial adjustment problems in adolescents, and specifically with CPV [2]. A relevant factor in the family sphere is the communication between parents and children, which is regarded as an indicator of the family climate’s quality [14,15]. When family communication is positive, cohesion in the family is strengthened because it fosters empathy, active listening, and support for children [16]. Conversely, offensive family communication, characterised by disrespectful and offensive language, reflects negative family functioning that is harmful to adolescents’ psychosocial development [17]. Recent works have highlighted the direct relationship between offensive family communication and CPV [18,19]. However, there is a scarce amount of research on other relevant individual psychosocial variables that are assumed to be involved in this relationship—such as those examined in this work.

At the individual level, several authors have observed that certain personal risk factors directly affect CPV such as low self-concept, narcissism, low empathy, and loneliness [20,21]. A factor that we consider relevant here and is today among the most widespread disorders in adolescent and young populations is psychological distress. The psychological distress construct encompasses depressive symptomatology, stress, and generalised anxiety disorder [22,23]. Previous studies have observed that the family climate is related to internalizing symptoms in youth [24,25]. Also, several authors have indicated that family communication problems are associated with psychological distress in children [26,27]. Moreover, other studies have equally observed that adolescents suffering from psychological distress are more likely to manifest violent behaviours towards their peers, e.g., violence towards their partners and cyberbullying [28,29]. In the case of CPV, Ibabe & Jaureguizar (2011) [30] found that children who assault their parents present high anxiety levels and, in a subsequent work, they encountered a significant relationship between CPV and depressive symptoms [31]. One possible explanation for the relationship between CPV and psychological distress is the fact that emotional and mental health problems are a risk factor in the development of problematic externalising behaviours such as violence [29,32].

Another major individual variable recently analysed in recent work on adolescent peer violence is the attitude towards institutional authority. This is divided into two dimensions: a positive attitude towards institutional authority, and a positive attitude towards the transgression of rules [33]. In this work, we have analysed only one of its two dimensions, the positive attitude towards the transgression of rules, and its relationship with CPV, which is a type of violence that occurs only in the family environment. In this sense, parents are the authority figures in the family and are the ones who impose and manage the rules of coexistence and the limits to children’s behaviour. The factors of the family relationship’s quality and family communication are associated with the attitude towards authority in adolescence [17,34]. More specifically, several studies have observed that problematic communication between parents and children enhances the development of adolescents’ transgressive attitude towards rules [32,35]. Moreover, studies on the role of transgressive behaviour in violent, disruptive, and criminal behaviours in adolescence have produced significant results [36,37]. Studies on CPV have also identified positive attitudes towards social norm transgression in adolescents as a CPV risk [38,39]. A further subject of interest in this study was to understand whether any gender differences existed in the relationships between the variables analysed. Previous studies on CPV have found that girls use verbal violence towards their parents more frequently than boys, who, for their part, resort more frequently to physical violence [39]. Differences in family communication have also been observed in other studies, with girls showing higher scores in offensive communication with their mothers than boys [40,41]. In addition, according to several studies, adolescent girls suffer more psychological distress than boys, which could be explained by girls’ greater emotional complexity in the face of relational problems [39,42,43,44,45]. However, boys have scored higher in the positive attitude towards social norm transgression, and girls show a greater positive attitude towards institutional authority [35,46,47].

### The Current Study

**Figure 1:**
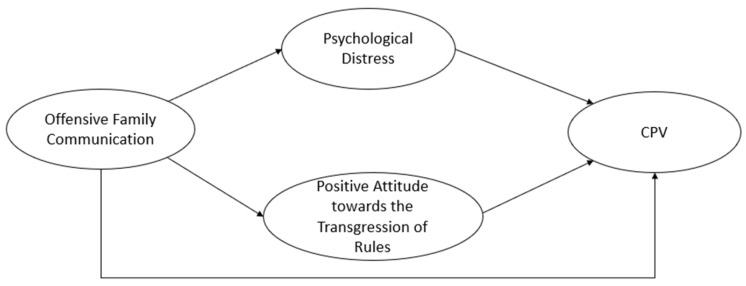
Theoretical model proposed (*Note*: CPV: child-to-parent violence).

Previous work has analysed the relationship of offensive family communication with other forms of violence in adolescence [18,48,49]. In a recent study by Romero-Abrio et al. [50] it was observed that offensive family communication is associated with cyberbullying, and that, in addition, there is also an indirect relationship between both factors moderated by psychological distress and a positive attitude towards the transgression of rules. The interest of the present study lies in examining how offensive family communication affects CPV, which differs from cyberbullying in that it is not a type of peer violence but rather from children towards parents. We consider it of interest to know the influence of the family environment in the genesis and development of the different manifestations of violent behaviour in adolescence. Therefore, the study objective was to analyse the relationship between family communication problems and CPV in adolescence, as well as the moderating role of psychological distress and a positive attitude towards social norm transgression in this relationship. The hypotheses were as follows (Figure 1): (1) offensive family communication is directly and significantly related to CPV; (2) offensive family communication is indirectly related to CPV, via psychological distress and a positive attitude towards the transgression of rules; and (3) these relationships present variations according to gender. 

## 2. Materials and Methods

### 2.1. Participants

The sample consisted of 7787 adolescents (51.5% boys and 48.5% girls) between 12 and 16 years of age (*M* = 13.37 and *SD* = 1.34) enrolled in secondary schools in the State of Nuevo León (Mexico), distributed in different age groups: 53.9% between 12 and 13 years of age, and 46.1% between 14 and 16 years of age. Participants were selected by proportional stratified sampling, and the geographic area was used as a sampling unit, resulting in 62.5% of adolescents being from urban schools and 37.5% from rural schools, of which 87.9% were public centres and 12.1% were private. Regarding grade level, 35.4% were enrolled in the first grade of secondary school, 33.9% in the second grade, and 30.7% in the third grade. A confidence level of 95% was used, with a sampling error of ±1.11, for *p* = *q* = 0.5. The response rate was 99.79% in this study, which was cross-sectional. The sample size has been calculated with a confidence level of 95%, a margin of error of 1.11% and with a proportion of *p* = *q* = 50%, giving us a sample of 7787 individuals. Missing scale or subscale data were treated using the multiple linear imputation model [51], always below 15%. Standardised scores were explored to identify univariate outliers [52,53]. 

### 2.2. Mesures

*Conflict Tactics Scale* [54]. In this study we used the adaptation made by [55], which consists of two subscales, one of violence towards the father and the other of violence towards the mother. Each contains six items referring to physical violence (e.g., “*I hit, punched, or slapped my parents*”) and verbal violence (e.g., “*I insult or have insulted or sworn at my parents*”). Item numbers 8 and 9, referring to economic violence, were excluded in this study. Each item has seven response options (from 0 = never to 7 = more than twenty times). The psychometric properties of the scale showed a good fit to the data: [SBχ^2^ = 11.1246, df = 7, *p* = 0.13328, CFI = 0.956, RMSEA = 0.017 (90% CI (0.000, 0.036)], NNFI = 0.912. The factor loadings varied. Cronbach’s alpha for the full scale was 0.71. For the subscale of violence towards the mother, it was 0.73, 0.77 (physical violence), and 0.72 (verbal violence); and for the subscale of violence towards the father, it was 0.76, 0.89 (physical violence), and 0.73 (verbal violence).

The *Parent–Adolescent Communication Scale* [56], later adapted by Jiménez et al. (2009, 2019) [18,57] was used. In the present study, we chose to use only the offensive communication subscale (e.g., “*My father/mother tries to offend me when he/she gets angry with me*”). In turn, this is divided into communication of the mother–adolescent, and communication of the father–adolescent. The subscale consists of ten items with five response options (0 = never, 5 = always). The subscale showed a good fit to the data; mother [SB χ^2^ = 2594.5748, df = 128, *p* < 0.001, CFI = 0.953, RMSEA = 0.049 (0.047, 0.051)]; father [SB χ^2^ = 2885.3985, df = 120, *p* < 0.001, CFI = 0.947, RMSEA = 0.053 (0.052, 0.055)]. Cronbach’s alpha was 0.77 (father) and 0.73 (mother).

*Psychological Distress Scale K10* [58]. The scale consists of ten items (e.g., “*How often did you feel restless or fidgety*”) and offers an overall score of psychological distress, with five response options (none of the time, a little of the time, some of the time, most of the time, and all of the time). The scale has been shown to have adequate psychometric properties: [SB χ^2^ = 504.7299, df = 29, *p* < 0.001, CFI = 0.981, RMSEA = 0.045 (0.042, 0.049)]. Factor loadings ranged between 0.68 and 0.74.

*Attitudes towards institutional authority in adolescents Scale (AAI-A)* [46]. In the present study, we opted for the subscale of a positive attitude towards the transgression of rules (e.g, “*It doesn’t matter if you break school rules if there are no punishments afterwards*”), which consists of four items with four response options (1 = totally disagree, 4 = totally agree). The scale showed a good fit to the data in the confirmatory scale analysis (CFA) [SB χ^2^ = 317.9209, df = 23, *p* < 0.001, CFI = 0.976, RMSEA = 0.040 (0.036, 0.044)]. Cronbach’s alpha was 0.75 for this subscale.

### 2.3. Procedure

The research was carried out within the framework of a collaboration between the Pablo de Olavide University (Spain) and the Autonomous University of Nuevo León (UANL) (Mexico). For data collection, the Secretary of Education in the State of Nuevo León called the inspectors of the various state regions to inform them of the start of this research. The inspectors informed their directors of the interest and relevance of the project, and requested them to grant access to the centres to researchers from the Faculty of Psychology of the UANL. In addition, staff from the Ministry of Education met with the parents of the selected schools to obtain informed consent. Once they were informed and permissions had been granted, the researchers from the UANL and the team of interviewers travelled to the randomly selected centres, in which the instruments were administered in three classrooms in each centre, one from each school grade. In order to avoid tiredness, the application of the questionnaire was carried out in two periods, with an 8 min break during which students were offered a snack to return to the classroom to continue answering the instrument. Also, the distribution of the scales was varied to avoid copying and a fatigue effect. Finally, to guarantee the confidentiality of the data, at the end of the questionnaire the student placed it in a sealed and slotted cardboard box. In those classrooms in which there were boys or girls with reading and comprehension problems, the administration was individualized by personnel trained at the UANL. All students were also informed of the objectives that were intended to be obtained with the research and that their participation was voluntary in such a way that if they did not wish to do so they could refuse to answer a question without entailing any type of sanction. The study complied with the ethical values required in human research [59]. The data was collected through a personal survey in the centres, and was carried out for two months, from September to October 2019.

### 2.4. Data Analysis

Firstly, Pearson correlations were calculated between all the variables studied and the *t*-test was performed. Then, an EQS 6.1 structural equations model [60] was calculated to analyse the relationship between the latent factors. Robust estimators were used to calculate the goodness-of-fit of the model and the statistical significance of the coefficients. IFC, IFI, and NNFI indices with values equal to or greater than 0.95 were considered acceptable, and for the RMSEA index, values equal to or less than 0.08. 

Once the model had been calculated with the general sample of adolescents, a multi-group analysis was carried out to examine the differences in the relationships obtained according to the sex of the adolescents. Two groups were established according to the sexes of the subjects: boys (N = 4102) and girls (N = 3375). Two models were calculated for the group of boys and girls, respectively. In the calculation of the first model (the restricted model), all relationships between the variables had to be equivalent. By contrast, the second model (the non-restricted model) was calculated without restrictions in the estimated parameters; hence, the relationships between the variables could differ in the different groups. Subsequently, the chi-square coefficient of the restricted and unrestricted models was compared; if this coefficient was significantly greater in the restricted model, invariance between the groups could be assumed.

## 3. Results

### 3.1. Pearsons Correlation

Table 1 shows the means, standard deviations, and correlations between the variables studied. The correlation analysis showed significant relationships between the variables studied, to see what the relationships are like between them and their degree of dependence. Of remarkable note were the relationships between the offensive communication towards the mother and psychological distress (r = 0.343, *p* < 0.01), the verbal violence towards the mother (r = 0.334, *p* < 0.01), the verbal violence towards the father (r = 0.244, *p* < 0.01) and the positive attitude towards the transgression of rules (r = 0.182, *p* < 0.01). Equally noteworthy were the relationships between the father’s offensive communication and verbal violence towards the father (r = 0.296, *p* < 0.01), psychological distress (r = 0.252, *p* < 0.01), verbal violence towards the mother (r = 0.233, *p* < 0.01) and the positive attitude towards the transgression of rules (r = 0.145, *p* < 0.01). Also remarkable were the relationships between the positive attitude towards the transgression of rules and verbal violence towards the mother (r = 0.222, *p* < 0.01) and verbal violence towards the father (r = 0.207, *p* < 0.01). Finally, the relationships between psychological distress and verbal violence towards the mother (r = 0.429, *p* < 0.01), and verbal violence towards the father (r = 0.357, *p* < 0.01) were particularly noteworthy.

### 3.2. Direct and Indirect Effects

**Figure 2:**
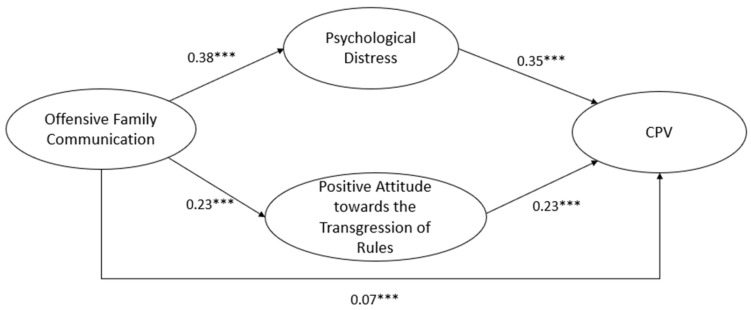
Final structural model with relationship coefficients and statistical significance. (*Note*: CPV: Child-to-parent violence). *** *p* < 0.001.

A structural equations model was then calculated with the EQS 6.0 program [53] (Table 2) and shows the latent variables included in the model, their respective indicators, the standard error, and the associated probability for each indicator in the corresponding latent variable, to see the importance of each dimension or subscale in the latent factor, which were previously set to one. Regarding the calculated equation model, the maximum likelihood method was used, with robust estimators (Mardia coefficient = 154.5272; Normalized estimator = 394.5511). The model showed an adequate fit to the data [SB χ^2^ = 370.3650; df = 39, *p* < 0.001; CFI = 0.98; RMSEA = 0.034 (0.031, 0.037)] and explained 28% of the variance in the CPV. As shown in Figure 2, the results indicated that offensive family communication was directly and positively related to CPV (these were estimated from the relationships (paths) between two variables or latent factors with one or several variables that mediate between them, β = 0.17, *p* < 0.001), to psychological distress (β = 0.38, *p* < 0.001), and to the positive attitude towards the transgression of rules (β = 0.23, *p* < 0.001). On the other hand, psychological distress was directly and positively related to CPV (β = 0.35, *p* < 0.001). Similarly, the positive attitude towards the transgression of rules was directly and positively related to CPV (β = 0.23, *p* < 0.001).

Regarding indirect effects (see Table 3), these were estimated from the relationships (paths) between two variables or latent factors with one or several variables that mediate between them. It was observed that offensive family communication was related to CPV through psychological distress (β = 0.131, CI [0.113–0.150], *p* < 0.001). Similarly, offensive family communication was related to CPV through the positive attitude towards the transgression of rules (β = 0.052, CI [0.041–0.063], *p* < 0.001).

### 3.3. Moderating Effect of Gender

In order to examine the existence of significant differences in the paths obtained, a multigroup analysis was performed. First, two groups were created according to the sexes of the individuals: boys (N = 4012) and girls (N = 3775). Next, two models were calculated, in which the first one imposes restrictions so that all the relationships between variables are the same; whereas, in the second model, no restrictions are imposed, so that differences in the relationships between variables can be found in the boys and the girls. Finally, the chi-square coefficient of both models was contrasted to see if invariance between the groups can be assumed. 

The results show significant differences in the model between adolescent boys and girls (Δχ^2^ (16, N = 7787) = 222.58; *p* < 0.001), and therefore, there is no metric invariance. Also, we added intercept constraints to the model comparison (scalar invariance), which do not show that there are statistically significant differences, and therefore, both measurement models are different and there is no scalar invariance (Δχ^2^ (11, N = 7787) = 395.083; *p* < 0.001). After examining the restricted model, it was decided to release eight restrictions that decreased the χ^2^ coefficient. Five restrictions refer to differences in the errors of the variables that make up the model, meaning that there is no substantial change in the relationships obtained in the general model. The other three relate to the relationship between the observable factors. It was found that the relationship between family communication problems and psychological distress is significant in the group of boys (β = 0.254; *p* < 0.001), but greater in the group of girls (β = 0.533; *p* < 0.001), while the relationships between family communication problems and child–parent violence and between family communication problems and positive attitudes towards violating rules are significant and greater for girls (β = 0.245, *p* < 0.001; β = 0.276, *p* < 0.001) than for boys (β = 0.100, *p* < 0.001; β = 0.222, *p* < 0.001). When the restrictions were removed, both models were shown to be equivalent for boys and girls (Δχ^2^ (8, N = 7787) = 13.38; *p* < 0.05).

## 4. Discussion

The study objective was to analyse the relationship between family communication problems and CPV, as well as the moderating role of psychological distress and a positive attitude regarding social norm transgression in this relationship, from an ecological perspective. As mentioned above, the ecological model of human development emphasizes the interrelationships between the individual characteristics of the subject and the contexts of socialization, which, in turn, are interconnected [13].

First, a direct and significant relationship was found between offensive family communication and CPV, confirming the first hypothesis. These data are consistent with those found in previous studies and emphasize, once again, the key role of positive family communication as a protective factor against violent behaviour in adolescents [19,57]. In addition, in the multigroup analysis, girls obtained higher scores in this relationship than boys, thus also partially confirming the third hypothesis. This result supports that of other studies in which this difference was specifically highlighted to be more widespread in mother–daughter communication. In addition, girls use more verbal violence against their mothers than physical violence [35,39]. Our results show that the mother’s offensive communication is significantly related to the child’s verbal violence towards their mother, with higher scores in the cases of the daughters. In this sense, our results provide empirical evidence of the fact that the mother–child relationship is a very significant factor in the regulation of violent child behaviour, especially that of daughters, and in particular in the case of CPV. The most likely explanation is that it consists of a response to the negative family climate in which they are immersed and in which they constantly perceive a lack of empathy and support from their parents [61]. They also find their mother figure to be a permanent source of conflict. This line of research should be explored more deeply in future works, delving into specific communication problems with mothers.

Second, our results showed an indirect relationship between offensive family communication and CPV through its relationships with psychological distress. This result partially confirms the second hypothesis and provides empirical evidence of the fact that, as observed in recent studies, offensive child–parent communication directly affects a child’s psychological distress [50,62]. Perceptions of a lack of parental support as well as offensive and disrespectful language towards children fosters negative feelings and emotions in adolescents. The latter then increases the possibility of anxiety and depressive symptoms. This psychological discomfort generated by family communication problems enhances, in turn, the appearance of CPV. The reason is probably a reaction against the hostile family climate and the need to achieve some degree of peace and harmony through behaviours such as verbal or physical force to reduce their levels of discomfort [63,64,65]. In addition, as hypothesised, results showed that girls obtained higher scores on the relationship between offensive family communication and psychological distress than boys. This finding is of great interest and consistent with that of recent studies according to which girls suffer more anxiety and show more depressive symptoms during adolescence [28,65]. They are also consistent with other studies that have observed gender differences in the relationship between the family climate and internalizing symptoms [66,67]. We can infer from our results that adolescent girls are more sensitive to a negative family climate and suffer more psychological problems than boys, probably because they also perceive and experience poor parental support as well as family relational and communication problems more intensely. The gender differences observed for these variables are highly relevant today and call for a more thorough analysis in future studies. 

Finally, on the other hand, an indirect relationship between offensive family communication and CPV through the positive attitude towards the transgression of rules was observed, confirming the second hypothesis. These results are compatible with those of previous studies according to which family communication problems are associated with children’s attitude towards institutional authority [17,37]. In families where family functioning is negative and parent–child communication is thus offensive and disrespectful, adolescents tend to transgress rules imposed both inside and outside the home [38]. Thus, children manifest their response to negative family functioning by adopting a defiant attitude towards their parents when they feel poorly understood and receive little affection [34,35,68]. This positive attitude towards norm transgression imposed on the family is, in turn, a risk factor for CPV, as reflected in the results of our study, confirming the recent observations of various researchers [38,39]. Our work shows that adolescents are more likely to use violence against their parents when they adopt transgressive behaviours towards social and family norms as a reaction to the deficiencies, especially of a communicative nature, that they perceive in their family functioning. One possible explanation is that children who are raised in hostile homes feel the need to defy established norms and figures of authority—in this case, their parents—and to engage in a visible power struggle with their parents [69,70]. In this situation, they use violence to measure their strength, lacking other more adaptive psychological and behavioural mechanisms that allow them to face family difficulties in a functional way. The multigroup analysis led to another significant result regarding gender: girls obtained higher scores than boys on the relationship between family communication problems and the positive attitude towards social norm transgression. We believe that this result is novel since it contrasts with the findings of other studies according to which adolescent boys are more likely to show positive attitudes towards social norm transgression than girls, who usually show a more positive attitude towards institutional authority [37,71]. Again, we observed that during their adolescence, girls are more sensitive to communication problems with their parents than boys and have few emotional resources to cope with a negative family climate. They adopt transgressive and rebellious behaviours towards their parents more often than boys do when they feel humiliated and disrespected by their parents, probably to minimise the negative feelings generated by the situation [39]. We consider it of great interest to pursue this subject in future studies. 

Lastly, this study presented some limitations. On the one hand, its cross-sectional design did not allow for the establishing of causal relationships between variables, so it would be relevant to expand the study with longitudinal research. On the other hand, the study is based solely on self-reported data: it did not consider any information of interest that could have been provided by parents and other significant agents of teenager socialisation, such as peers or teachers. This may have a direct impact on the external validity of the research, as it implies a type of bias in data collection. Broader sources of information should be incorporated in future studies on CPV to pursue the same line of analysis and reflection. In addition, the sample selected included more subjects who attended urban schools than rural schools. This imbalance in the sample, in relation to this socio-geographical factor, may lead to a bias in the results obtained. Furthermore, the age range of 16 to 20 years has not been analysed, which may constitute another potential bias since the study did not observe the entire stage of adolescence. It would be interesting to expand the research with a sample that includes late adolescence. Finally, another possible research bias may be due to negative environmental conditions, which may cause adolescents to report different responses when they know they are being observed. In relation to the external validity of the study, caution must be taken in the generalization of the results. The selected sample is very large but the presence of the potential biases described above may limit the extent of the results.

## 5. Conclusions

From a psychosocial perspective, child-to-parent violence is a complex subject to study. Many social, familial, and individual factors are involved in the genesis and development of child-to-parent violence. This type of violence takes place within the family sphere and many parent narratives contain biases or conceal information, making it therefore difficult to collect objective data. In addition, novel forms of child-to-parent violence are emerging today in our changing society, such as the use of technological means to exercise violence (child-to-parent cyberviolence) [6]. We are also witnessing new family models (single-parent, reconstituted, or homoparental families, etc.) which are essential to study to better understand CPV. In short, we must pursue our study of child-to-parent violence in order to generate new approaches and to further our understanding of the phenomenon. Our work offers interesting keys to guide psychoeducational intervention towards the prevention of CPV in the family context: training in positive parenting and non-violent communication, family conflict resolution, and the prevention of problems related to mental health in adolescence.

In future work, it would be interesting to delve more deeply into the gender perspective in CPV, and other factors related to adolescent socialization, perhaps in newer contexts, such as the virtual context. Similarly, it would be interesting to conduct more studies that relate CPV to other types of violence, such a dating violence, and also, to widen the age range to be over 16 years of age. As mentioned above, there is diversity in families in the present day, and studies should be extended to single-parent families, homosexual families, and regrouped families, as they are increasingly present in society.

## Figures and Tables

**Table 1:** Correlations, means, and standard deviations.

Variables	1	2	3	4	5	6	7	8
1. Positive Attitude towards the Transgression of Rules	1							
2. Psychological Distress	0.130 **	1						
3. Physical Violence Towards the Mother	0.149 **	0.175 **	1					
4. Verbal Violence Towards the Mother	0.222 **	0.429 **	0.396 **	1				
5. Physical Violence Towards the Father	0.150 **	0.141 **	0.647 **	0.279 **	1			
6. Verbal Violence Towards the Father	0.207 **	0.357 **	0.314 **	0.676 **	0.439 **	1		
7. Offensive Communication of the Mother	0.182 **	0.343 **	0.157 **	0.334 **	0.127 **	0.244 **	1	
8. Offensive Communication of the Father	0.145 **	0.252 **	0.099 **	0.233 **	0.132 **	0.296 **	0.691 **	1
M/SD	1.58/0.068	2.04/0.85	1.06/0.27	1.56/0.66	1.07/0.30	1.45/0.64	2.02/0.78	1.93/0.79

*Notes*: ** *p* < 0.01.

**Table 2:** Parameter estimates, standard errors, and associated probability.

Variables	Factorial Loading General Model
Offensive Family Communication	
Mother’s Offensive Communication	5.195 (0.112) ***
Father’s Offensive Communication	3.998 (0.100) ***
Psychological Distress	
Part 1 of Psychological Distress	2.911 (0.040) ***
Part 2 of Psychological Distress	2.469 (0.34) ***
Part 3 of Psychological Distress	2.469 (0.034) ***
Positive Attitude Towards the Transgression of Rules	
Item 4	0.440 (0.015) ***
Item 8	0.649 (0.015) ***
Item 9	0.725 (0.014) ***
Item 10	0.608 (0.015) ***
Child-to-Parent Violence	
Child-to-parent Violence Mother	2.631 (0.092) ***
Child-to-parent Violence Father	2.263 (0.092) ***

*Notes*: Robust statistics. Standard errors are in parentheses. Factors were set to 1 robust statistic. Standard errors are in parentheses. Factors were set to 1. *** *p* < 0.001.

**Table 3:** Indirect, direct, and total effects of the overall model.

	β	Standard Errors (s)	*p*	95% CI	
				LCL	UCL
Indirect effects					
OFC → PD → CPV	0.131	0.009	<0.001	0.113	0.150
OFC → PATTR → CPV	0.052	0.006	<0.001	0.041	0.063
Direct effects					
OFC → CPV	0.173	0.019	<0.001	0.136	0.210
Total effects					
OFC → CPV	0.357	0.019	<0.001	0.306	0.408

*Notes*: OFC: Offensive Family Communication; PD: Psychological Distress; PATTR: Positive Attitude Towards the Transgression of Rules; CPV: Child-to-Parent Violence.

## Data Availability

Data are available upon request.

## References

[1] Pereira R., Loinaz Calvo I., Bilbao H., Arrospide J., Bertino L., Calvo A., Gutiérrez M.M. (2017). Propuesta de definición de violencia filio-parental: Consenso de la Sociedad Española para el estudio de la Violencia filio-parental (SEVIFIP). Papeles Psicólogo—Psychol. Pap..

[2] Suárez-Relinque C., Arroyo G.d.M., León-Moreno C., Jerónimo J.E.C. (2019). Child-to-parent violence: Which parenting style is more protective? A study with spanish adolescents. Int. J. Environ. Res. Public Health.

[3] Cottrell B. (2003). Parent Abuse: The Abuse of Parents by Their Teenage Children.

[4] Arias-Rivera S., García V.H. (2020). Theoretical framework and explanatory factors for child-to-parent violence. A scoping review. An. Psicol..

[5] Contreras L., Rodríguez-Díaz F.J., Cano-Lozano M.C. (2020). Prevalence and reasons for child-to-parent violence in Spanish adolescents: Gender differences in victims and aggressors. Colección Psicología y Ley.

[6] Suarez Relinque C., Moral-Arroyo G. (2023). del Child-to-parent cyber violence: What is the next step?. J. Fam. Violence.

[7] Lyons J., Bell T., Fréchette S., Romano E. (2015). Child-to-Parent Violence: Frequency and Family Correlates. J. Fam. Violence.

[8] Gallego R., Novoa M., Fariña F., Arcea R. (2019). Child-to-parent Violence and Parent-to-child Violence: A Meta-analytic Review. Eur. J. Psychol. Appl. Leg. Context.

[9] Calvete E., Orue I., Fernández-González L., Chang R., Little T.D. (2020). Longitudinal Trajectories of Child-to-Parent Violence through Adolescence. J. Fam. Violence.

[10] Ibabe I. (2019). Adolescent-to-parent violence and family environment: The perceptions of same reality?. Int. J. Environ. Res. Public Health.

[11] Rico E., Rosado J., Cantón-Cortés D. (2017). Impulsiveness and Child-to-Parent Violence: The Role of Aggressor’s Sex. Span. J. Psychol..

[12] Bronfenbrenner U. (1994). Ecological models of human development. International Encyclopedia of Education.

[13] Soyer G.F. (2019). Book Review: The Ecology of Human Development by Urie Bronfenbrenner. J. Cult. Values Educ..

[14] Hereyah Y., Purwanti A. (2021). Family Communication on Single-Parenting Families in Maintaining Relationships and Shaping Children’s Self-Concepts. Ilkogr. Online.

[15] Jiménez T., Murgui S., Musitu G. (2007). Comunicación familiar y ánimo depresivo: El papel mediador de los recursos psicosociales del adolescente. Rev. Mex. Psicol..

[16] Olson D.H. (2000). Circumplex Model of Marital and Family Sytems. J. Fam. Ther..

[17] Castro-Castañeda R., Núñez-Fadda S.M., Musitu G., Callejas-Jerónimo J.E. (2019). Comunicación con los padres, malestar psicológico y actitud hacia la autoridad en adolescentes mexicanos: Su influencia en la victimización escolar. Estud. Sobre Educ..

[18] Jiménez T.I., Estévez E., Velilla C.M., Martín-Albo J., Martínez M.L. (2019). Family Communication and Verbal Child-to-Parent Violence among Adolescents: The Mediating Role of Perceived Stress. Int. J. Environ. Res. Public Health.

[19] López-Martínez P., Montero-Montero D., Moreno-Ruiz D., Martínez-Ferrer B. (2019). The Role of Parental Communication and Emotional Intelligence in Child-to-Parent Violence. Behav. Sci..

[20] Cortina H., Martín A.M. (2020). The behavioral specificity of child-to-parent violence. An. Psicol..

[21] González-Álvarez M., Morán Rodríguez N., García-Vera M.P. (2011). Violencia de hijos a padres: Revisión teórica de las variables clínicas descriptoras de los menores agresores. Psicopatol. Clin. Leg. Forense.

[22] Cummings C.M., Caporino N.E., Kendall P.C. (2014). Comorbidity of anxiety and depression in children and adolescents: 20 years after. Psychol. Bull..

[23] Keles B., McCrae N., Grealish A. (2020). A systematic review: The influence of social media on depression, anxiety and psychological distress in adolescents. Int. J. Adolesc. Youth.

[24] Rea H.M., Factor R.S., Kao W., Shaffer A. (2020). A meta-analytic review of the five minute speech sample as a measure of family emotional climate for youth: Relations with internalizing and externalizing symptomatology. Child Psychiatry Hum. Dev..

[25] Holfeld B., Baitz R. (2020). The mediating and moderating effects of social support and school climate on the association between cyber victimization and internalizing symptoms. J. Youth Adolesc..

[26] Curran T., Allen J. (2017). Family Communication Patterns, Self-Esteem, and Depressive Symptoms: The Mediating Role of Direct Personalization of Conflict. Commun. Rep..

[27] Schrodt P., Ledbetter A.M. (2007). Communication processes that mediate family communication patterns and mental well-being: A mean and covariance structures analysis of young adults from divorced and nondivorced families. Hum. Commun. Res..

[28] Cénat J.M., Hébert M., Blais M., Lavoie F., Guerrier M., Derivois D. (2014). Cyberbullying, psychological distress and self-esteem among youth in Quebec schools. J. Affect. Disord..

[29] Morelli M., Bianchi D., Baiocco R., Pezzuti L., Chirumbolo A. (2016). Sexting, psychological distress and dating violence among adolescents and young adults. Psicothema.

[30] Ibabe I., Jaureguizar J. (2011). ¿Hasta qué punto la violencia filio-parental es bidireccional?. An. Psicol..

[31] Ibabe I., Arnoso A., Elgorriaga E. (2014). Behavioral problems and depressive symptomatology as predictors of child-to-parent violence. Eur. J. Psychol. Appl. Leg. Context.

[32] Romero-Abrio A., Martínez-Ferrer B., Sánchez-Sosa J.C., Musitu G. (2019). A psychosocial analysis of relational aggression in Mexican adolescents based on sex and age. Psicothema.

[33] Emler N., Reicher S. (1995). Adolescence and Delinquency: The Collective Management of Reputation.

[34] Estévez E., Murgui S., Moreno D., Musitu G. (2007). Estilos de comunicación familiar, actitud hacia la autoridad institucional y conducta violenta del adolescente en la escuela [Family communication styles, attitude towards institutional authority and violent behavior of adolescents in school]. Psicothema.

[35] Carrascosa L., Cava M.J., Buelga S. (2015). Actitudes hacia la autoridad y violencia entre adolescentes: Diferencias en función del sexo [Attitudes towards authority and violence among adolescents: Differences according to sex]. Suma Psicológ..

[36] Emler N., Reicher S. (2005). Delinquency: Cause or consequence of social exclusion. The Social Psychology of Inclusion and Exclusion.

[37] Ortega-Barón J., Buelga S., Caballero M.J.C., Torralba E. (2017). School violence and attitude toward authority of student perpetrators of cyberbullying. Rev. Psicodidact..

[38] Del Moral G., Suárez-Relinque C., Callejas J.E., Musitu G. (2019). Child-to-parent violence: Attitude towards authority, social reputation and school climate. Int. J. Environ. Res. Public Health.

[39] Martínez-Ferrer B., Romero-Abrio A., Moreno-Ruiz D., Musitu G. (2018). Child-to-parent violence and parenting styles: Its relations to problematic use of social networking sites, alexithymia, and attitude towards institutional authority in adolescence. Psychosoc. Interv..

[40] Buelga S., Martínez-Ferrer B., Musitu G. (2015). Family relationships and cyberbullying. Cyberbullying Across the Globe: Gender, Family, and Mental Health.

[41] Buelga S., Martínez–Ferrer B., Cava M.J. (2017). Differences in family climate and family communication among cyberbullies, cybervictims, and cyber bully–victims in adolescents. Comput. Hum. Behav..

[42] Garaigordobil M. (2009). A comparative analysis of empathy in childhood and adolescence: Gender differences and associated socio-emotional variables. Int. J. Psychol. Psychol. Ther..

[43] Brewer G., Kerslake J. (2015). Cyberbullying, self-esteem, empathy and loneliness. Comput. Hum. Behav..

[44] Garaigordobil M., Martinez-Valderrey V., Aliri J. (2013). Autoestima, empatía y conducta agresiva en adolescentes víctimas de bullying presencial. Eur. J. Investig. Health Psychol. Educ..

[45] Levant R.F., Hall R.J., Williams C.M., Hasan N.T. (2009). Gender Differences in Alexithymia. Psychol. Men Masculinity.

[46] Cava M.J., Estévez E., Buelga S., Musitu G. (2013). Propiedades psicométricas de la escala de actitudes hacia la autoridad institucional en adolescentes (AAI-A). An. Psicol..

[47] Espelage D.L. (2014). Ecological Theory: Preventing Youth Bullying, Aggression, and Victimization. Theory Pract..

[48] Cava M.J., Buelga S., Musitu G. (2014). Parental communication and life satisfaction in adolescence. Span. J. Psychol..

[49] López E.E., Olaizola J.H., Ferrer B.M., Ochoa G.M. (2006). Aggressive and non-aggressive rejected students: An analysis of their differences. Psychol. Sch..

[50] Romero-Abrio A., Martínez-Ferrer B., Musitu-Ferrer D., León-Moreno C., Villarreal-González M.E., Callejas-Jerónimo J.E. (2019). Family communication problems, psychosocial adjustment and cyberbullying. Int. J. Environ. Res. Public Health.

[51] Sarstedt M., Ringle C.M., Hair J.F. (2017). Partial Least Squares Structural Equation Modeling. Handbook of Market Research.

[52] Batista-Foguet J.M., Coenders G. (2000). Modelos de Ecuaciones Estructurales: Modelos Para el Análisis de Relaciones Causales.

[53] Bentler P.M. (2006). EQS 6 Structural Equations Program Manual.

[54] Straus M.A., Douglas E.M. (2004). A Short Form of the Revised Conflict Tactics Scales, and Typologies for Severity and Mutuality. Violence Vict..

[55] Del Hoyo-Bilbao J., Orue I., Gámez-Guadixb M., Calvete E. (2020). Multivariate models of child-to-mother violence and child-to-father violence among adolescents. Eur. J. Psychol. Appl. Leg. Context.

[56] Barnes H., Olson D., Olson D.H. (1982). Parent adolescent communication scale. Family Inventories.

[57] Jiménez T.I., Musitu G., Ramos M.J., Murgui S. (2009). Community involvement and victimization at school: An analysis through family, personal and social adjustment. J. Community Psychol..

[58] Kessler R., Mroczek D. (1994). Final Version of Our Non-Specific Psychological Distress Scale.

[59] World Medical Association Declaration of Helsinki (2013). World Medical Association Declaration of Helsinki Ethical Principles for Medical Research Involving Human Subjects. J. Am. Med. Assoc..

[60] Hair J.F., Black W.C., Babin B.J., Anderson R.E. (2009). Structural Equation Modeling Basics. Multivariate Data Analysis.

[61] Buelga S., Iranzo B., Postigo J., Carrascosa L., Ortega Barón J. (2018). Parental Communication and Feelings of Affiliation in Adolescent Aggressors and Victims of Cyberbullying. Soc. Sci..

[62] Clements-Nolle K., Waddington R. (2019). Adverse childhood experiences and psychological distress in juvenile offenders: The protective influence of resilience and youth assets. J. Adolesc. Health.

[63] Kowalski R.M., Limber S.P. (2013). Psychological, physical, and academic correlates of cyberbullying and traditional bullying. J. Adolesc. Health.

[64] Iranzo B., Buelga S., Cava M.J., Ortega-Barón J. (2019). Cyberbullying, psychosocial adjustment, and suicidal ideation in adolescence. Psychosoc. Interv..

[65] Van Droogenbroeck F., Spruyt B., Keppens G. (2018). Gender differences in mental health problems among adolescents and the role of social support: Results from the Belgian health interview surveys 2008 and 2013. BMC Psychiatry.

[66] Hughes E.K., Gullone E. (2008). Internalizing symptoms and disorders in families of adolescents: A review of family systems literature. Clin. Psychol. Rev..

[67] Schulz S., Nelemans S.A., Oldehinkel A.J., Meeus W., Branje S. (2021). Examining intergenerational transmission of psychopathology: Associations between parental and adolescent internalizing and externalizing symptoms across adolescence. Dev. Psychol..

[68] Musitu G., Estévez E., Emler N. (2007). Adjustment problems in the family and school contexts, attitude towards authority, and violent behavior at school in adolescence. Adolescence.

[69] Mazzone A., Camodeca M. (2019). Bullying and Moral Disengagement in Early Adolescence: Do Personality and Family Functioning Matter?. J. Child Fam. Stud..

[70] Nuñez-Fadda S.M., Castro-Castañeda R., Vargas-Jiménez E., Musitu-Ochoa G., Callejas-Jerónimo J.E. (2020). Victimization among Mexican Adolescents: Psychosocial Differences from an Ecological Approach. Int. J. Environ. Res. Public Health.

[71] Jiménez T.I., Estévez E., Murgui S. (2014). Ambiente comunitario y actitud hacia la autoridad: Relaciones con la calidad de las relaciones familiares y con la agresión hacia los iguales en adolescentes. An. Psicol..

